# Screening and Molecular Identification of Bacteria from the Midgut of *Amphimallon solstitiale* Larvae Exhibiting Antagonistic Activity against Bacterial Symbionts of Entomopathogenic Nematodes

**DOI:** 10.3390/ijms222112005

**Published:** 2021-11-05

**Authors:** Marcin Skowronek, Ewa Sajnaga, Waldemar Kazimierczak, Magdalena Lis, Adrian Wiater

**Affiliations:** 1 Laboratory of Biocontrol, Production and Application of EPN, Centre for Interdisciplinary Research, The John Paul II Catholic University of Lublin, 20-708 Lublin, Poland; ewa.sajnaga@kul.pl (E.S.); waldemar.kazimierczak@kul.pl (W.K.); magdalena.lis@kul.pl (M.L.); 2 Department of Industrial and Environmental Microbiology, Institute of Biological Sciences, Maria Curie-Sklodowska University, 20-033 Lublin, Poland

**Keywords:** midgut microbiota, bacterial interactions, entomopathogenic nematodes, *Xenorhabdus*, *Photorhabdus*, *Amphimallon solstitiale*

## Abstract

Entomopathogenic nematodes (Rhabditida: *Steinernematidae* and *Heterorhabditidae*) are a group of organisms capable of infecting larvae of insects living in soil, including representatives of the family *Scarabaeidae*. Their insecticidal activity is related to the presence of symbiotic bacteria *Xenorhabdus* spp. or *Photorhabdus* spp. in the alimentary tract, which are released into the insect body, leading to its death caused by bacterial toxins and septicemia. Although the antibacterial activities of symbionts of entomopathogenic nematodes have been well described, there is insufficient knowledge of the interactions between these bacteria and microorganisms that naturally inhabit the alimentary tract of insects infested by nematodes. In this study, 900 bacterial strains isolated from midgut samples of *Amphimallon solstitiale* larvae were tested for their antagonistic activity against the selected five *Xenorhabdus* and *Photorhabdus* species. Cross-streak tests showed significant antibacterial activity of 20 isolates. These bacteria were identified as *Bacillus [Brevibacterium] frigoritolerans*, *Bacillus toyonensis*, *Bacillus wiedmannii*, *Chryseobacterium lathyri*, *Chryseobacterium* sp., *Citrobacter murliniae*, *Enterococcus malodoratus*, *Paenibacillus* sp., *Serratia marcescens* and *Serratia* sp. Since some representatives of the intestinal microbiota of *A. solstitiale* are able to inhibit the growth of *Xenorhabdus* and *Photorhrhabdus* bacteria in vitro, it can be assumed that this type of bacterial interaction may occur at certain stages of insect infection by *Steinernema* or *Heterorhabditis* nematodes.

## 1. Introduction

Representatives of the genera *Photorhabdus* and *Xenorhabdus* (γ-Proteobacteria, *Morganellaceae*) occur naturally in two types of environments. One of them is the intestinal lumen of free-living third-stage juveniles of soil-inhabiting entomopathogenic nematodes (EPNs). *Photorhabdus* species form mutualistic relationships with nematodes of the genus *Heterorhabditis*, while *Xenorhabdus* are symbionts of nematodes from the genus *Steinernema* [1]. Infective juveniles actively seek out suitable hosts, i.e., larvae of insects from the *Scarabaeidae* family, in the surface soil layer and enter their bodies through natural openings (mouth, anus, tracheae, or spiracles) [2]. After entering the insect, nematodes release bacteria through regurgitation (*Heterorhabditis*) or defecation (*Steinernema*) into the hemocoel [3]. Initially, this cavity and next the entire larval cadaver become another habitat of nematode-associated bacteria. *Photorhabdus* and *Xenorhabdus* bacteria produce numerous enzymes that decompose insect tissues, e.g., proteases, lipases, hemolysins, and chitinases, which enriches the environment with nutrients thereby supporting rapid growth of bacteria and nematodes [4]. EPN bacteria can dominate this habitat, as they are able to produce numerous bacteriocins and other substances with antibacterial properties [5,6,7].

Although the activities of nematode symbionts during infection have been quite well explored, there is almost no knowledge about the mutual relationships between these bacteria and microorganisms constituting the insect gut microbiota. Noteworthy, such interactions must be considerably limited in the early stage of infection, since nematodes that have entered the insect’s gut through natural openings from the external environment penetrate the hemocoel and release their bacterial symbionts in this body area. Therefore, there is a physical barrier separating the gut microbiota from the nematode-associated bacteria developing in the hemolymph. However, this barrier leaks as nematodes penetrate and damage the intestine. It has been found that, during the early phase of infection of *Manduca sexta* by *Steinernema carpocapsae*, many species of gut microbes are translocated into the hemocoel, where they are initially present together with *Xenorhabdus nematophila* released by nematodes [8]. It is only between 18 and 24 h after the onset of infection that *X. nematophila* becomes quantitatively dominant, while bacterial species originating from the insect gut gradually disappear, although some (in particular *Enterococcus faecalis*) are relatively resistant to *X. nematophila* antibiotics and can be found in the hemolymph even after 48 h.

Noteworthy, the gut wall in insects infested with EPNs is not only leaky due to the damage induced by the nematode but also unstable, as bacteria released by nematodes produce enzymes that decompose insect tissues, including the wall of the digestive system. It has been found that, already at a very early stage of the *Photorhabdus luminescens* infection of *M. sexta* larvae, bacteria begin their growth in a region between the basal lamina and the midgut epithelium cells. In these conditions, *Photorhabdus* express a number of toxins (e.g., gut-active Toxin complex A (Tca) and an RTX-like metalloprotease PrtA). This induces massive programmed cell death of the midgut epithelium [9]. Similarly, *Photorhabdus temperata* is able to attach to the same space underneath the basal lamina in the midgut epithelium of the sugarcane stalk borer *Diatraea saccharalis*. It is suggested that the function of the *P. temperata* subpopulation that migrates to the midgut tissue is to inhibit the main source of immediate competitors, i.e., the midgut luminal microbiota [10]. In some cases, direct contact between nematode-associated and insect bacteria may occur early during the nematode infection, since it has been shown that nematodes can release their bacterial symbionts to the intestine, not reaching the hemocoel. Using GFP-labeled *X. nematophila* bacteria, it was found that some *S. carpocapsae* infective juveniles released its bacterial symbionts already in the alimentary tract of *Spodoptera littoralis* larvae [11].

Summarizing, the midgut microbiota of insects infected by EPNs can potentially interact with bacterial nematode symbionts at various stages of infection. Since some insect gut bacteria are capable of inhibiting the growth of *Xenorhabdus* or *Photorhabdus* bacteria, their activity may be one of the lines of defense of the insect host against nematode infection. Elucidation of the interactions between antagonistic groups of bacteria during EPN infection may provide a more complete picture of its course, which will help to understand insect defense mechanisms against nematode attack.

In this study, we describe screening of bacterial strains from *A. solstitiale* midgut for antibacterial activity against five EPN symbionts of the genera *Xenorhabdus* and *Photorhabdus*. Although the presence of strains with such activities in the alimentary tract of insect larvae does not fully prove the occurrence of this type of interactions in vivo, it certainly makes this phenomenon highly probable.

## 2. Results

### 2.1. Cross-Streak Tests

**Figure 1:**
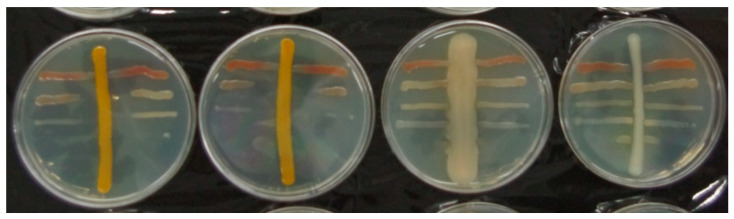
Cross-streak tests between *A. solstitiale* midgut isolates and EPN symbionts. Horizontal streaks–*Xenorhabuds* or *Photorhabdus*, vertical streak–midgut isolate. Plates on the left-isolates with high antibacterial activity against EPN bacteria strains (*Chryseobacterium* sp.); plates on the right-no inhibition effect.

At the beginning of the study, 150 bacterial strains were randomly isolated from midguts sampled from 10 *A. solstitiale* individuals (L2 and L3 larvae freshly collected from the natural environment). Next, potential antibacterial properties of these isolates were investigated using cross-streak tests against the following EPN bacteria species: *Xenorhabdus bovienii*, *X. budapestensis*, *X. kozodoii*, *X. nematophila*, and *P. temperata* (Figure 1). Four isolates (denoted as AT4 L2 01, AT5 L2 01, AT5 L2 02, and AT5 L2 04) were able to inhibit the growth of the selected bacteria species (Table 1). Another 750 bacterial strains were isolated from the midguts of larvae that had survived the 12-day exposure to EPNs. The aim of the use of midguts of larvae subjected to this type of pressure was to increase the probability of obtaining isolates able to inhibit the growth of nematode symbions. 66% of insect larvae exposed to IJ of *Heterorhabditis megidis* as well as 57%, 73%, 63%, and 60% of larvae exposed to *Steinernema arenarium*, *S. bicornutum*, *S. carpocapsae*, and *S. kraussei*, respectively, survived the EPNs exposure. The determination of the antibacterial activity of the isolates with the use of cross-streak tests showed that the highest number of strains inhibiting the growth of the selected EPN bacteria strains (11) was isolated from the midgut of larvae subjected to the exposure to *S. arenarium*. Additionally, three isolates originating from larvae exposed to *S. kraussei* and two from larvae exposed to *H. megidis* were positive (Table 1).

In the cross-streak test, some isolates from the midguts of *A. solstitiale* larvae were able to completely inhibit the growth of the selected nematode symbionts over the entire surface of Petri dishes. None of these bacteria completely inhibited the growth of all EPN bacteria strains used in the cross-test, but strain ATK3 L3 01 was able to inhibit the growth of 4 out of the 5 species. *X. bovienii* and *X. nematophila* were the most sensitive to the antimicrobial activity of the isolates. The growth of *X. bovienii* and *X. nematophila* was completely inhibited by 12 and 9 isolates, respectively. In turn, *P. temperata* was characterized by the lowest susceptibility to growth inhibition. Only 5 isolates were able to prevent the growth of these bacteria, and the inhibition zones were significantly smaller than those of *Xenorhabdus* bacteria.

### 2.2. Maximum Inhibitory Dilution Tests

**Figure 2:**
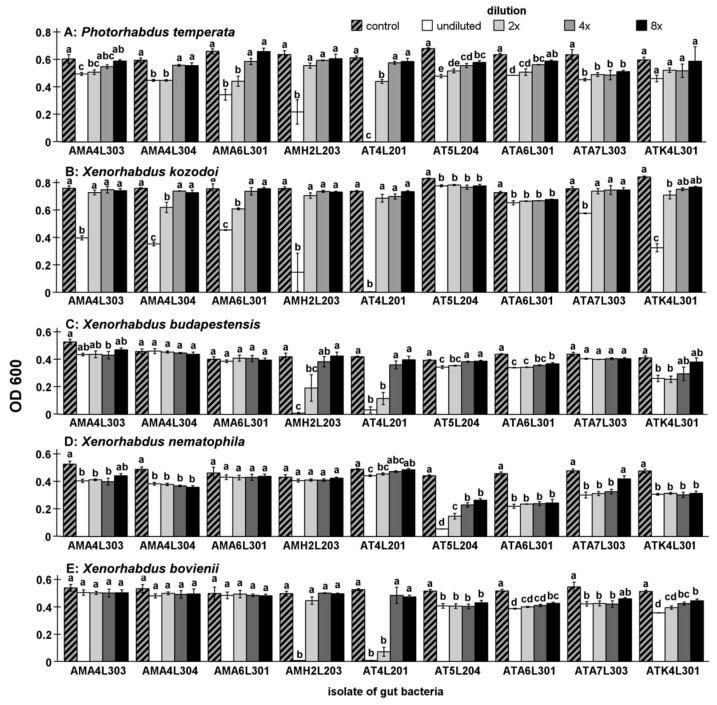
Differences in the antibacterial activities of increasing dilutions of supernatants from selected *A. solstitiale* gut isolates against *P. temperata* (**A**), *X. kozodoi* (**B**), *X. budapestensis* (**C**), *X. nematophila* (**D**) and *X. bovienii* (**E**) in the MID tests. The same letters above bars in the group mean no statistically significant differences between the inhibitory activity of supernatants.

The next stage of the study consisted in assessment of the ability of post-culture filtrates of isolates selected in the previous stage to inhibit EPN bacteria growth. Maximum inhibitory dilution (MID) tests were used for this purpose. The tests showed the EPN bacteria inhibitory ability of post-culture filtrates of 9 isolates from *A. solstitiale* midguts (Figure 2). In most cases, however, only partial growth inhibition of only some species (most often *X. kozodoii*) was recorded. Complete or almost complete inhibition of the growth of several EPN bacteria species was detected only in the case of undiluted filtrates from 2 isolates, i.e., AT4 L2 01 and AMH2 L2 03.

The results of the MID tests were statistically analyzed (one-way ANOVA, Tukey’s HSD test). The supernatant dilution of all (except ATK L3 01) isolateshad a significantly influenced *P. temperata* growth (ANOVA, F_14,4_ ≥ 7.201, *p* < 0.005). The undiluted supernatant of the AT4 L2 01 isolate completely inhibited the growth of *P. temperata* (*t*-test, *t*_4_ = 46.315, *p* < 0.001). The undiluted supernatant of the AMA4 L3 03 isolate exerted the lowest inhibitory effect on *P. temperata* growth (*t*-test *t*_4_ = 3.299, *p* < 0.05).

The supernatant dilution of all the isolates had a statistically significant influence on *X. kozodoi* growth (ANOVA, F_14,4_ ≥ 7.202, *p* < 0.05). The undiluted supernatant of the AT4 L2 01 isolate caused almost complete inhibition of *X. kozodoi* growth (*t*-test, *t*_4_ = 137.756, *p* < 0.001). In the undiluted supernatant group AMH2 L2 03 isolate exerted the lowest inhibitory effect on *X. kozodoi* growth (*t*-test *t*_4_ = 4.377, *p* < 0.05).

The supernatant dilution of AMH2 L2 03, AT4 L2 01, AT5 L2 04, ATA6 L3 01 and ATK L3 01 had a statistically significant influence on *X. budapestensis* growth (ANOVA, F_14,4_ ≥ 5.584, *p* < 0.05). The undiluted supernatant of the AMH2 L2 03 isolate caused almost complete inhibition of *X. budapestensis* growth (*t*-test, *t*_4_ = 14.935, *p* < 0.001). In the undiluted supernatant group, the supernatant of the AMA4 L3 04 isolate exerted no inhibitory effect on *X. budapestensis* growth (*t*-test *t*_4_ = 0.127, *p* > 0.05).

The supernatant dilution of all the isolates, except AMA6 L3 01 and AMH2 L2 03, had a statistically significant influence on *X. nematophila* growth (ANOVA, F_14,4_ ≥ 8.026, *p* < 0.05). In the undiluted supernatant group, the supernatant of the AMA6 L3 01 isolate exerted no inhibitory effect on *X. nematophila* growth (*t*-test *t*_4_ = 0.682, *p* > 0.05).

### 2.3. Molecular Identification of Isolates

All isolates showing the ability to limit the growth of EPN microsymbionts in the cross-streak experiments were taxonomically identified with the use of gene sequence analysis. The first step of molecular identification was based on 16S rDNA sequencing. Almost full-length sequences of the 16S rRNA gene were determined by PCR performed with the use of universal primers. Subsequently, the *rpoB* or *gapA* gene sequences were determined using primers specific to a given genus (Table 2).

The 16S rRNA threshold values of 95% and 98.7% gene sequence similarity between the tested isolate and the reference strain were used as indicators that an isolate represented a certain genus or species, respectively. The affiliation of an isolate to the given species was considered finished when the 16S rRNA gene sequence analysis were in agreement with those based on the *rpoB* or *gapA* sequencing. As shown in Table 3, the strains shared very high 16S rRNA gene sequence similarity (98.4–100%) to some type strain sequences deposited in GenBank, indicating that they represented genera *Bacillus* (7 isolates), *Chryseobacterium* (6 isolates), *Citrobacter* (2 isolates), *Enterococcus* (2 isolates), *Serratia* (2 isolates)*,* and *Paenibacillus* (1 isolate). Then, on the basis of the *rpoB* or *gapA* sequence identity values, we identified 4 isolates as *Chryseobacterium lathyri*, 3 isolates as *Bacillus wiedmannii*, 2 isolates as *Bacillus toyonensis*, 2 isolates as *Bacillus [Brevibacterium] frigoritolerans*, 2 isolates as *Enterococcus malodoratus*, 2 isolates as *Citrobacter murliniae*, and 1 isolate as *Serratia marcescens*. These identification results were finally confirmed by phylogenetic analysis based on 16S rDNA, and *rpoB* or *gapA* markers, demonstrating close relationships of the tested analyzed isolates with the reference strains reflected in formation of well separated branches with high bootstrap values. Only 4 isolates could not be assigned to the species level, as they were clearly separated from the recognized species (Supplementary Data Figures S1—S3).

## 3. Discussion

A comparison of the data presented in Table 1 and Figure 2 shows that the majority of the *A. solstitiale* midgut isolates capable of inhibiting EPN bacteria growth in the cross-tests did not exhibit such potential in the MID tests. The post-culture filtrates of only 9 of the 20 isolates indicated by the cross-tests had antimicrobial activity against the selected nematode symbionts. Similar results were presented in our previous study, where midgut bacteria were isolated from *M. melolontha*. Only 7 of the 38 isolates selected with the use of the cross-tests exhibited antimicrobial activity in the MID tests [17]. Probably, the antagonistic activity of some midgut isolates against EPN symbionts is induced by direct contact with these bacteria. Hence, in the absence of such contact in the MID tests, no EPN bacteria growth inhibition was observed in many cases. Noteworthy, the strains with the highest antimicrobial activity in the MID tests (AT4 L2 01 and AMH2 L2 03) did not exhibit the highest level of this activity in the cross-tests. Based on the results of the investigations of bacteria colonizing the midguts of *A. solstitiale* and *M. melolontha* larvae, it can be concluded that cross-tests seem to be a simple and effective method for screening insect midgut strains capable of inhibiting EPN bacteria growth, whereas MID tests have quite limited application in this case. The agar well diffusion tests and agar disk diffusion tests, whose suitability was checked in our research, failed as well. In the vast majority of cases, these tests showed no antimicrobial activity of the tested isolates, probably for the same reason as in the case of the MID tests.

The genotyping approach based on 16S rRNA gene is helpful for taxonomic identification of bacterial isolates, especially as there are currently accepted sequence similarity threshold values available established at 98.65% and 95% for species and genus delineation, respectively [18,19,20]. However, the 16S rRNA gene is not recommended as the only molecular marker for effective differentiation of bacterial species, as high conservation, recombination events, and horizontal gene transfer of rRNA genes may lead to misinterpretation in species classification [21,22,23]. Therefore, comparable sequence analysis of 16S rRNA verified by analysis of single housekeeping gene sequences have been used in many studies for fast, relatively cheap, and reliable identification of bacteria derived from diverse environments [13,24,25]. The chosen housekeeping gene should meet the criteria of a single copy in the genome, high discriminatory power, and no proneness to recombination and lateral gene transfer [26]. By sequencing the 16S rRNA together with *rpoB* or *gapA*, which are molecular markers with proven effectiveness [15,25,27], we were able to provide an accurate species identification for 16 of the 20 isolates. The other 4 isolates were attributed to a certain genus, potentially representing new species.

The most numerous isolates were representatives of the genera *Bacillus* and *Chryseobacterium*. *Bacillus* spp. have been isolated from the alimentary tract of many *Scarabaeidae* species. They were isolated e.g., from the midgut of *Melolontha hippocastani* larvae [28]. The presence of *Bacillus licheniformis* exhibiting cellulase activity was detected in hindguts of *Holotrichia parallela* larvae [29]. Cellulose-decomposing *Bacillus* bacteria were also isolated from the midgut of unidentified *Scarabaeidae* larvae collected in the tropical forests of Costa Rica [30]. The presence of *Bacillus* bacteria was also detected in the midguts of non-scarab beetles, e.g., *Dendroctonus armandi* [31], *Agrilus planipennis* [32], and *Poecilus chalcites* [33]. One of the strains described in this study, *Bacillus toyonensis*, together with several insect-pathogen bacterial species, such as *Bacillus cereus*, *Bacillus anthracis*, and *Bacillus thuringiensis*, are included in the “*Bacillus cereus* group”, i.e., a group of species with closely related phylogeny [34]. Two other isolates were identified as *B. wiedmannii*, a species that is also closely related to this group of bacilli; hence, it has been proposed that they should be included in this group [35]. Some members of this group, e.g., *B. thuringiensis* and *B. cereus*, are well known pathogens of insects. They enter the organism via the alimentary system, i.e., they are temporarily present in the midgut. *B. thuringiensis* was isolated from *M. melolontha* larvae [36,37], while *B. cereus* was detected in *A. solstitiale* larvae [38]. However, no pathogenicity of *B. toyonensis* or *B. wiedmannii* against insects has been reported to date. In addition to the bacteria from the genus *Bacillus*, one of the isolates was identified as a representative of the phylogenetically close genus *Paenibacillus*. *P. popilliae*, which is a known insect pathogen, has been repeatedly detected in *Scarabaeidae* larvae, e.g., in the hemolymph of *Amphimallon solstitiale* [39] and *Popillia japonica* [40].

Bacteria of the genus *Chryseobacterium* have quite often been found in the organisms of beetles, e.g., in the midgut of *Protaetia brevitarsis* [41], *D. armandi* [31], and *A. planipennis* [32]. In contrast to *Bacillus* and *Paenibacillus* bacteria, no pathogenicity of *Chryseobacterium* bacteria against *Scarabaeidae* larvae has been reported to date.

The next four isolates are representatives of the genera *Citrobacter* and *Serratia* from the order *Enterobacterales*. *Citrobacter* bacteria have been isolated from midguts of *Scarabaeidae* larvae, e.g., *H. parallela* [29] and *Lepidiota mansueta* [42]. The presence of *Serratia* bacteria has been detected in *Scarabaeidae* larvae as well. *S. marcescens* and *S. liquefaciens* have been isolated from the hemocoel of *M. melolontha* larvae [43]. Noteworthy, both bacterial species have been found to be pathogenic to *Dendroctonus micans*, *Thaumetopoea pityocampa*, and *Lymantria dispar* larvae. *Serratia* spp. have been isolated from midguts of *M. hippocastani* larvae [28] and *Scarabaeidae* larvae collected in Costa Rica [30].

The issue of the antagonistic activity of insect midgut microbiota against symbiotic bacteria of EPNs has been investigated to a very limited extent to date. Wollenberg et al. [44] isolated two *Stenotrophomonas* strains from cadavers of *Galleria mellonella* larvae that were previously infested with *H. megidis*. These bacteria were resistant to *Photorhabdus*-produced antibiotics and were able to inhibit the growth of some *Photorhabdus* strains. The obtained results suggested that the *Stenotrophomonas* species secreted a heat-stable molecule with inhibitory activity against *Photorhabdus* strains. More examples of the antagonistic activities of insect gut bacteria can be found in our previous study, in which we described some isolates from midguts of *M. melolontha* larvae capable of inhibiting the growth of EPN bacteria [17]. We identified 12 active isolates, half of which were *Pseudomonas chlororaphis* strains, and the others were classified as *C. murliniae*, *Acinetobacter calcoaceticus*, *C. lathyri*, *Chryseobacterium* sp., *S. liquefaciens*, and *Serratia* sp. As can be seen, the results of the previous study are partially consistent with the present report. *C. murliniae*, *C. lathyri*, and *Serratia* bacteria exhibiting antimicrobial activity against *Xenorhabdus* and *Photorhabdus* were isolated from the midguts of both *M. melolontha* and *A. solstitiale*. Taking into account the species richness of the midgut microbiota in *Scarabaeidae* larvae, the isolation of the same or related bacterial species in separate studies from larvae of two different insect species does not seem to be accidental. This probably indicates that these bacterial species or genera, compared to other bacteria inhabiting larval midguts, are more efficient in inhibition of the growth of pathogenic nematode symbionts and can be their natural antagonists. In addition to the bacterial species isolated from the midguts of both insect species, some bacteria were present in only one species. Especially interesting is the absence of *P. chlororaphis* among the isolates obtained from *A. solstitiale*, as the bacteria constituted a significant percentage of all active isolates from *M. melolontha* and its strains had the highest ability to inhibit *Xenorhabdus* and *Photorhabdus* growth. In turn, there were 7 strains representing the genus *Bacillus* among the active isolates obtained from *A. solstitiale*, while this genus was not detected among the *M. melolontha* isolates. However, this does not prove the specificity of bacteria exhibiting antagonistic properties against EPN bacterial symbionts in the midguts of larvae of the studied *Scarabaeidae* species. This was contradicted by our additional nanopore-sequencing studies of the midgut microbiota of *M. melolontha* and *S. solstitilae* larvae, but these results were only partially published [45]. The investigations demonstrated the presence of *B. frigoritolerans* in 17 of 31 studied midgut samples from *A. solstitiale* and 13 of 32 midgut samples from *M. melolontha*. *B. wiedmannii* was detected in 18 samples from *A. solstitiale* and 22 samples from *M. melolontha.* In turn, *B. toyonensis* was present in 16 samples from *A. solstitiale* and 15 samples from *M. melolontha.* Similarly, in the case of all other species isolated from *A. solstitiale* or *M. melolontha* midguts, showing antimicrobial activity against nematode bacterial symbionts, the metataxonomic studies showed their presence in both *Scarabaeidae* species (data not shown). The failure to isolate the same bacterial species from the midguts of both insects was most probably associated with the insufficient scale of our research, although it is also likely that not all bacterial strains of species identified in the presented study are capable of EPN bacteria growth inhibition.

Although some of the bacterial strains reported in this study had not been shown previously to exert an antagonistic effect on other bacterial species, such properties are not surprising in many cases. *Bacillus* spp. are known to produce a broad spectrum of antimicrobial substances, (peptide/lipopeptide antibiotics, bacteriocins) [46,47]. Culture extracts of *B. toyonensis* strain isolated from *Folsomia candida* gut had high inhibitory activity against *Staphylococcus aureus*, *Pseudomonas syringae*, *Micrococcus luteus*, and *Bacillus subtilis*. Genome analysis revealed several gene clusters coding the production of bacteriocins [48]. Similarly, Lopes et al. [49] sequenced the whole *B. toyonensis* BAC3151 genome and found that it had a higher frequency of putative bacteriocin gene clusters than that of *Bacillus* species used traditionally for production of antimicrobials. In turn, secondary metabolite extracts produced by *B. toyonensis* collected from sea waters inhibited the growth of *Vibrio alginolyticus*, *Aeromonas hydrophila*, and *Pseudomonas aeruginosa* [50].

*Enterococcus* bacteria are also well known to produce bacteriocins, referred to as enterocins in this case. Although no such antimicrobial peptides have been found in *E. malodoratus* to date, numerous enterocins have been detected in *Enterococcus faecium*, *Enterococcus faecalis*, or *Enterococcus mundtii* [51,52].

The greatest number of the active isolates obtained in this study was classified as the genus *Chryseobacterium*. Bacteria of this genus are not widely known for their antibacterial properties. Nevertheless, the antimicrobial properties of *Chryseobacterium antibioticum* against *P. aeruginosa* and *Escherichia coli* have been described [53], while bacteriocin gene clusters have been detected in *Chryseobacterium indologenes* [54].

Antibacterial activities have been described in the case of *Serratia* and *Citrobacter* bacteria as well. A *S. marcescens* strain originating from *M. melolontha* produced a bacteriocin-like substance with high activity against *B. thuringiensis*, *B. cereus*, *Enterobacter* sp., *E. faecalis*, *Proteus mirabilis*, *Pseudomonas fluorescens*, *Staphylococcus cohni*, *Staphylococcus epidermidis*, and many phytopathogenic bacteria [55]. Another *S. marcescens* strain was reported to produce bacteriocin 28b with activity against *E. coli* [56]. In turn, *S. marcescens* strain ZPG19 produced prodigiosin, i.e., a bioactive secondary metabolite with antimicrobial effects [57]. In the case of *Citrobacter* bacteria, no antibacterial properties of *C. murliniae* have been described yet, while it has been reported that a bacteriocin from *Citrobacter freundii* is able to inhibita wide range of Gram-negative bacteria [58] and *Citrobacter* sp. produces antibacterial lipopeptides inhibiting *S. aureus* growth [59].

The study results show the presence of numerous bacterial strains in the midgut of *A. solstitiale* larvae with potential to inhibit the growth of *Xenorhabdus* or *Photorhabdus* bacteria introduced into the insect’s organism by EPNs. Some of these strains have already been demonstrated to have antibacterial activity, although not against EPN bacteria. However, our study does not answer the key question whether the interactions also occur in vivo and may constitute one of the insect defense mechanisms. Elucidation of this issue would be highly important. It should also be noted that, although a relatively large number of midgut isolates were analyzed in our research (900 strains from *A. solstitiale* and 900 strains from *M. melolontha* isolated previously), many bacterial strains of interest have certainly not been detected. The selection procedures applied in the study allowed identification of only some bacterial species present in the larval midgut with a capability of strong antagonistic interactions against *Xenorhabdus* and *Photorhabdus* bacteria. Therefore, these fragmentary results require further comprehensive research to provide a complete picture of this problem.

## 4. Materials and Methods

### 4.1. EPNs Culturing and Maintenance

The study involved 5 EPN species isolated from soil sampled in Poland and identified using molecular methods. The nematodes were reproduced in *G. mellonella* (Lepidoptera: *Pyralidae*) larvae, grown on a natural diet and weighing 180–200 mg. Before use in the experiments, infective juveniles of EPNs were kept in a sterile aqueous solution at 8 °C for no longer than 15 days.

### 4.2. EPN Bacterial Symbionts

In this study 5 species of EPN symbionts were isolated and used: X. bovienii (host: S. kraussei), X. nematophila (host: S. carpocapsae), X. kozodoii (host: S. arenarium), X. budapestensis (host: S. bicornutum), and *P. temperata* (host: H. megidis). Cultures of the bacteria were suspended in lysogeny broth (LB) supplemented with 20% glycerol and placed at −85 °C. Before use in the experiments thawed stock cultures of bacteria were tested on the NBTA plates to check the colony form. Only blue or green (primary form) colonies were used in subsequent analyses.

### 4.3. Source of A. solstitiale Larvae

The *A. solstitiale* larvae used in the study were collected in the Lublin region (East Poland). Larvae that were not subjected to nematode pressure and those exposed to *S. arenarium*, *S. bicornutum*, and *P. temperata* were found in grasslands located within the administrative boundaries of the city of Lublin, while the other larvae were collected in forest nursery plots located in Kozłowieckie Forests. The collected white grubs were placed separately in 50-mL plastic vessels with moist soil to protect the insects from stress (drying, biting). Only healthy L2 or L3 larvae were used in the experimental and control groups.

### 4.4. Exposure of A. solstitiale to Selected EPN Species

White grubs of *A. solstitiale* were placed individually on the bottom of 150-mL vessels containing 100 g of moist (~−50 kPa water potential) soil originating from the harvesting field. Each insect larva was exposed to ~1000 IJs of EPN. The cups with the scarab larvae were stored in incubators for 12 days at 20 °C.

### 4.5. Isolation of Bacteria from A. solstitiale Midgut

All larvae were surface sterilized using 70% alcohol and washed twice in sterile water. After drying for 1 min, the digestive tract was dissected. Next, the midgut was homogenized in 1 mL of 0.5% NaCl in a glass tissue grinder. Appropriate dilutions were spread on LB agar plates and incubated at 20 °C for 2–4 days in aerobic or microaerobic (6% oxygen) conditions. Single colonies were transferred to agar slants for further analyses.

### 4.6. Antimicrobial Activity Assays

The antimicrobial activity was evaluated by cross-streak tests and MID tests as described previously [17]. The only difference was the centrifugation parameters of the culture fluids used for the MID tests, which were as follows: 12,000 rpm (about 10,000× *g*) for 15 min.

### 4.7. DNA Extraction and PCR

The genomic DNA was isolated from 20 bacterial strains using a Genomic Mini AX Bacteria Spin Kit (A&A Biotechnology, Gdynia, Poland) in accordance with the manufacturer instruction. All amplification reactions were carried out with FirePol Master Mix (Solis BioDyne, Tartu, Estonia). PCR amplifications of 2 molecular markers for each isolate, i.e., the 16S *rRNA* gene and *rpoB* (coding RNA polymerase subunit β) or *gapA* (coding glyceraldehyde-3 phosphate dehydrogenase) were performed using primer pairs and PCR cycling parameters described in Table 2. Purifications of the amplicons were performed with the use of Clean-Up Kit (A&A Biotechnology, Gdynia, Poland). The amplicons were sequenced using the same primers in Genomed S.A. (Warsaw, Poland).

### 4.8. Gene Sequencing and Phylogenetic Analysis

The gene sequences of the isolates were analyzed using nucleotide BLAST (NCBI). Additionally, the 16S rRNA gene sequences were compared with those included in the EzBioCloude database [60]. The determined single gene sequences were aligned using ClustalW [61] implemented in the MEGA 6.06 software [62]. Sequence identity values were estimated using BioEdit 7.2 [63]. The phylograms were constructed using the neighbor-joining (NJ) method [64] in the Maximum Composite Likelihood model in Mega 6.06. Bootstrap values for individual nodes demonstrating tree robustness were calculated for 1000 replicates [65].

The gene sequences obtained were submitted to the GenBank under accession numbers shown in Table 3 and on the phylogenetic trees (Figures S1–S3).

### 4.9. Statistical Analysis

As no statistically significant differences were found between the replicates of the experiments, the data were pooled together before statistical analysis. The normalcy of the data was checked by the Shapiro-Wilk test. The homogeneity of variance was assessed by the Levene test. MID tests results were subjected to one-way ANOVA. Means were separated with the Tukey HSD test. For pairwise comparisons (bacteria species) the t-student test was used. Differences among the means were considered significant at *p* < 0.05. All of the statistical analyses were performed using the SPSS Statistics 27 software package (IBM Corp., Armonk, NY, USA).

## Figures and Tables

**Table 1:** Cross-streak tests between selected *A. solstitiale* midgut isolates and five EPN symbionts.

Strain ID *	Size of Inhibition Zones (mm) Left Inhibition Zone|Right Inhibition Zone				
	*P. temperata*	*X. kozodoii*	*X. bovienii*	*X. nematophila*	*X. budapestensis*
AT4 L2 01	0|0	0|0	26|23	no gr.**|no gr.	no gr.|22
AT5 L2 01	9|8	11|12	no gr.|no gr.	no gr.|no gr.	23|no gr.
AT5 L2 02	5|7	9|10	no gr.|no gr.	no gr.|28	12|12.
AT5 L2 04	0|0	9|9	no gr.|no gr.	21|no gr.	12|12
ATA3 L3 05	2|2	9|6	no gr.|no gr.	27|no gr.	22|19
ATA6 L3 01	0|0	0|0	20|19	13|14	0|0
ATA6 L3 02	0|0	0|0	23|no gr.	no gr.|no gr.	0|0
ATA7 L3 01	0|0	0|0	no gr.|no gr.	22|no gr.	0|0
ATA7 L3 02	0|0	9|11	21|23	32|no gr.	15|9
ATA7 L3 03	0|0	0|0	22|23	18|25	0|0
ATA7 L3 04	0|0	10|11	no gr.|no gr.	22|no gr.	12|12
AMA4 L3 03	0|0	17|18	no gr.|no gr.	no gr.|no gr.	27|30
AMA4 L3 04	0|0	16|19	no gr.|no gr.	no gr.|no gr.	24|25
AMA6 L3 01	0|0	0|0	no gr.|no gr.	no gr.|no gr.	28|no gr.
AMA7 L3 01	4|6	0|0	7|8	7|7	3|3
ATK3 L3 01	0|0	no gr.|no gr.	no gr.|no gr.	no gr.|no gr.	no gr.|no gr.
ATK4 L3 01	0|0	7|6	no gr.|no gr.	no gr.|no gr.	no gr.|no gr.
AMK6 L3 01	0|0	0|0	no gr.|no gr.	11|14	28|14
ATH3 L2 10	0|0	16|18	25|21	no gr.|no gr.	no gr.|25
AMH2 L2 03	1|1	8|6	12|12	6|7	12|13

* Strain ID coding: 2nd letter in strain ID: M–bacteria isolated in microaerobic conditions; T–bacteria isolated in aerobic conditions. 3rd letter in strain ID: *A. solstitiale* larvae exposed to: A–*S. arenarium*; K–*S. kraussei*; H–*H. megidis*. L2 or L3: developmental stage of the nsect larva. ** no gr.–no growth (complete inhibition of bacterial strain growth).

**Table 2:** Oligonucleotides used in the study.

Primer	Sequence	Target Gene	Target Bacterial Genus	PCR Cycling	Product Length	Reference
27F 1492R	5′-AGAGTTTGATCCTGGCTCAG-3′ 5′-GGTTACCTTGTTACGACTT-3′	16S rDNA	All tested	3 min 95 °C, 30 × (30 s 94 °C, 45 s 55 °C, 90 s 72 °C), 5 min 72 °C	1500 bp	[12]
rpoB1206 rpoBR3202	5-′ATCGAAACGCTGAAGGTCCAAACAT-3′ 5′-ACACCCTTGTTACCGTGACGACC-3′	*rpoB*	*Bacillus*	3 min 95 °C, 35 × (20 s 94 °C, 30 s 55 °C, 90 s 72 °C), 5 min 72 °C	1170 bp	[13]
ESbre-rpoF ESbre-rpoR	5′-GTTTTGGACCTTCCGAATCTGA-3′ 5′-TGGGCGTAGACGCTCATAGAT-3′	*rpoB*	*Bacillus* *[Brevibacterium]*	3 min 95 °C, 30 × (60 s 94 °C, 30 s 50 °C, 60 s 72 °C), 5 min 72 °C	660 bp	This work
ESchr-rpoF ESchr-rpoR	5′GGTGAAGTAGTTTCTATCGAAAGA-3′ 5′-ATGTTTGGTCCTTCCGGAGTT-3′	*rpoB*	*Chryseobacterium*	3 min 95 °C, 30 × (35 s 95 °C, 35 s 52 °C, 50 s 72 °C), 5 min 72 °C	790 bp	This work
Vic3 Vic2	5′-GGCGAAATGGCWGAGAACCA-3′ 5′-GAGTCTTCGAAGTTGTAACC-3′	*rpoB*	*Citrobacter*	4 min 94 °C, 30 × (30 s 94 °C, 30 s 50 °C, 45 s 72 °C), 5 min 72 °C	410 bp	[14]
ESent-rpoF ESent-rpoR	5′-AACGAAGGTGTTGTTGAATTCGT-3′ 5′-CGAAACGTTGTCCACCAAATTG-3′	*rpoB*	*Enterococcus*	3 min 95 °C, 30 × (60 s 94 °C, 30 s 50 °C, 60 s 72 °C), 5 min 72 °C	1170 bp	This work
F1 R1	5′-GTDAAARTDGGTATTAACGGHTTYGG-3′ 5′-TTGTCRTACCARGMWAYRRYTTTHACCA-3′	*gapA*	*Paenibacillus*	3 min 95 °C, 30 × 30 s 95 °C, 30 s 55 °C, 50 s 72 °C), 5 min 72 °C	900 bp	[15]
359f 359r	5′-TTATCGCTCAGGCGAACTCCAAC-3′ 5′-TGCTGGATTCGCCTTTGCTACG-3′	*rpoB*	*Serratia*	3 min 95 °C, 30 × (50 s 94 °C, 40 s 52 °C, 60 s 72 °C), 5 min 72 °C	530 bp	[16]

**Table 3:** Molecular identification of bacterial strains with antimicrobial activity isolated from the midgut of *A. solstitiale* larvae.

Strain ID	Identification Result/Gene Accession Numbers	Strain with the Highest Similarity to the Isolate in the Gene Bank Based on the Gene Sequence/Gene Accession Number/% Nucleotide Identity	
		16S rDNA	rpoD/Gap *
AT4 L2 01	*Citrobacter murliniae* 16S rDNA-MW468102 *rpoB*-MW481653	*C. braaki* ATCC 51113^T^ (NAEW01000064) 99.6% *C. europeus* 97/99^T^ (FLYB01000015) 99.6% *C. murliniae* CDC 2970-59T^T^ (AF025369) 99.6%	*C. murliniae* CIP 104556^T^ (KM516007) 99.7% *C. braakii* CIP 104554^T^ (KF057930) 96.5%
AT5 L2 01	*Bacillus toyonensis* 16S rDNA-MW467541 *rpoB*-MW481644	*B. mobilis* 0711P9-1^T^ (KJ812449) 100% *B. thuringensis* ATCC 10792^T^ (ACNF01000156) 100% *B. toyonensis* BCT-712^T^ (CP006863) 99.9%	*B. toyonensis* BCT-7112^T^ (NC_022781) 99.3% *B. mobilis* 0711P9^T^ (NZ_MACH01000024) 98.8%
AT5 L2 02	*Enterococcus* *malodoratus* 16S rDNA-MW468100 *rpoB*-MW481650	*E. malodoratus* ATCC 43197T (ASWA01000002) 99.8% *E. gilvus* ATCC BAA-350T (AJDQ01000009) 99.7%	*E. malodoratus* ATCC 43197^T^ (HQ611251) 99.2% *E. gilvus* ATCC BAA-350^T^ (HQ611245) 90.7%
AT5 L2 04	*Enterococcus* *malodoratus* 16S rDNA-MW468101 *rpoB*-MW481651	*E. malodoratus* ATCC 43197^T^ (ASWA01000002) 99.8% *E. gilvus* ATCC BAA-350^T^ (AJDQ01000009) 99.7%	*E. malodoratus* ATCC 43197^T^ (HQ611251) 99.2% *E. gilvus* ATCC BAA-350^T^ (HQ611245) 90.7%
ATA3 L3 05	*Citrobacter murliniae* 16S rDNA *rpoB*-MW557598	*C. braaki* ATCC 51113^T^ (NAEW01000064) 99.6% *C. europeus* 97/99^T^ (FLYB01000015) 99.6% *C. murliniae* CDC 2970-59T^T^ (AF025369) 99.6%	*C. murliniae* CIP 104556^T^ (KM516007) 99.7% *C. braakii* CIP 104554^T^ (KF057930) 96.5%
ATA6 L3 01	*Bacillus wiedmannii* *16S rDNA*-MW467540 *rpoB*-MW481647	*B. wiedmannii* FSL W80169^T^ (KU198626) 99.9% *B. proteolitycus* TD42^T^ (MACH0000033) 99.8%	*B. wiedmannii* FSL W80169^T^ (LOBC01000027) 99.4% *B. proteolitycus* TD42^T^ (NZ_MACH01000136) 96.0%
ATA6 L3 02	*Chryseobacterium* sp. 16S rDNA-MM429323 *rpoB*-MN445060	*C. viscerum* 687B-08^T^ (FR871426) 98.6% *C. tructae* 1084-08^T^ (FR871429) 98.4%	*C. jejuense* DSM 19299^T^ (JX293153) 96.8% *C. nakagawai* NCTC13529^T^ 96.6% *C. viscerum* 687B-08^T^ (PEG02000009) 95.6%
ATA7 L3 01	*Bacillus wiedmanniii* 16S rDNA-MW467539 *rpoB*-MW481646	*B. proteolitycus* TD42^T^ (MACH0000033) 100% *B. toyonensis* BCT-7112^T^ (CP006863) 100% *B. wiedmannii* FSL W8-0169^T^ (KU198626) 99.9%	*B. wiedmannii* FSL W80169^T^ (LOBC01000027) 99.4% *B. toyonensis* BCT-7112^T^ (NC_022781) 96.8% *B. proteolitycus* TD42^T^ (NZ_MACH01000136) 96.0%
ATA7 L3 02	*Chryseobacterium* *lathyri* 16S rDNA-MM429324 *rpoB*-MN445061	*C. lathyri* RBA2-6^T^ (DQ673674) 99.8%	*C. lathyri* KCTC 22544^T^ (NZ_QNFY01000004) 99.1%
ATA7 L3 03	*Serratia sp.* 16S rDNA-MM422008 *rpoB*-MN445054	*S. plymuthica* DSM 4540^T^ (AJ233433) 99.6%	*Serratia quinivorans* LMG 7887^T^ (JX425320) 98.3% *Serratia plymuthica* LMG 7886^T^ (JX425330) 98.1%
ATA7 L3 04	*Chryseobacterium* *lathyri* 16S rDNA-MM429320 *rpoB*-MN445062	*C. lathyri* RBA2-6^T^ (DQ673674) 99.8%	*C. lathyri* KCTC 22544^T^ (NZ_QNFY01000004) 98.4
AMA4 L3 03	*Chryseobacterium* *lathyri* 16S rDNA-MM429319 *rpoB*-MN445053	*C. lathyri* RBA2-6^T^ (DQ673674) 99.4%	*C. lathyri* KCTC 22544^T^ (NZ_QNFY01000004) 98.6%
AMA4 L3 04	*Chryseobacterium* *lathyri* 16S rDNA-MM429321 *rpoB*-MN445057	*C. lathyri* RBA2-6^T^ (DQ673674) 99.2%	*C. lathyri* KCTC 22544^T^ (NZ_QNFY01000004) 98.6%
AMA6 L3 01	*Chryseobacterium* sp. 16S rDNA-MM429322 *rpoB*-MN445059	*C. viscerum* 687B-08^T^ (FR871426) 98.7% *C. tructae* 1084-08^T^ (FR871429) 98.5%	*C. jejuense* DSM 19299^T^ (JX293153) 96.8% *C. nakagawai* NCTC13529^T^ (LR134386) 96.4% *C. viscerum* 687B-08^T^ (PEG02000009) 95.4%
AMA7 L3 01	*Bacillus wiedmanniii* 16S rDNA-MW467543 *rpoB*-MW481643	*B. mobilis* 0711P9^T^ (KJ812449) 99.7% *B. toyonensis* BCT-7112^T^ (CP006863) 99.7% *B. wiedmannii* FSL W80169^T^ (KU198626) 99.6%	*B. wiedmannii* FSL W80169^T^ (LOBC01000027) 99.7% *B. mobilis* 0711P9^T^ (NZ_MACH01000024) 99.1% *B. toyonensis* BCT-7112^T^ (NC_022781) 97.0%
ATK3 L3 01	*Bacillus* *[Brevibacterium]* *frigoritolerans* 16S rDNA-MW467545 *rpoB*-MW481648	*B. frigoritolerans* DSM 8801^T^ (AM747813) 99.5% *B. simplex* NBRC 15720^T^ (NR_042136) 99.1%	*B. frigoritotolerans* FJAT-2396^T^ (NZ_KV440950) 98.3% *B. simplex* DSM1321^T^ (CP017704) 96.8%
ATK4 L3 01	*Bacillus* *[Brevibacterium]* *frigoritolerans* 16S rDNA-MW467544 *rpoB*-MW481649	*B. frigoritolerans* DSM 8801^T^ (AM747813) 99.5% *B. simplex* NBRC 15720^T^ (DSM1321T) 99.2%	*B. frigoritotolerans* FJAT-2396^T^ (NZ_KV440950) 98.3% *B. simplex* DSM1321^T^ 96.8% (CP017704)
AMK6 L3 01	*Bacillus toyonensis* 16S rDNA-MW467542 *rpoB*-MW481645	*B. mobilis* 0711P9^T^ (KJ812449) 99.7% *B. toyonensis* BCT-712^T^ (CP006863) 99.5%	*B. toyonensis* BCT-7112^T^ (NC_022781) 100% *B. mobilis* 0711P9^T^ (NZ_MACH01000024) 97.5%
ATH3 L2 10	*Serratia marcescens* 16S rDNA-MW468103 *rpoB*-MW481652	*S. marcescens* subsp. *marcescens* ATCC 13880^T^ (IMPQ01000005) 99.6%	*S. marcescens* subsp. *marcescens* ATCC 13880^T^ (CP041233) 97.3%
AMH2 L2 03	*Paenibacillus* sp. 16S rDNA-MW468104 *gap*- MW481654	*P. peoriae* DSM 8320^T^ (AJ320494) 99.8% *P. jamilae* KACC 10925^T^ (QVP01000089) 99.6% *P. polymyxa* ATCC 842^T^ (AFOX01000032) 99.4%	*P. jamilae* KACC 10925^T^ (NZ_QVPU01000020) 95.9% *P. cribbensis* AM49^T^ (CP020028) 95.8% *P. peoriae* KCTC 3763^T^ (NZ_AGFX01000038) 94.5%

* The *rpoB* gene was analyzed for all strains mentioned, except *Peanibacillus* sp., for which the *gapA* gene was analyzed.

## Data Availability

Not applicable.

## Supplementary Materials

The following are available online at https://www.mdpi.com/article/10.3390/ijms222112005/s1.
