# Supplementary material for: Screening and Molecular Identification of Bacteria from the Midgut of Amphimallon solstitiale Larvae Exhibiting Antagonistic Activity against Bacterial Symbionts of Entomopathogenic Nematodes
Source: Int J Mol Sci. 2021 Nov 5;22(21):12005. doi: 10.3390/ijms222112005 (PMC8584744; doi:10.3390/ijms222112005)
Supplement: Supplementary file 1 [file ijms-22-12005-s001.zip › ijms-1425227-supplementary.pdf]

Supplementary data

Article: Screening and Molecular Identification of Bacteria from the Midgut of *Amphimallon solstitiale* Larvae Exhibiting Antagonistic Activity Against Bacterial Symbionts of Entomopathogenic Nematodes

Authors: Marcin Skowronek, Ewa Sajnaga, Waldemar Kazimierczak, Magdalena Lis and Adrian Wiater

A)

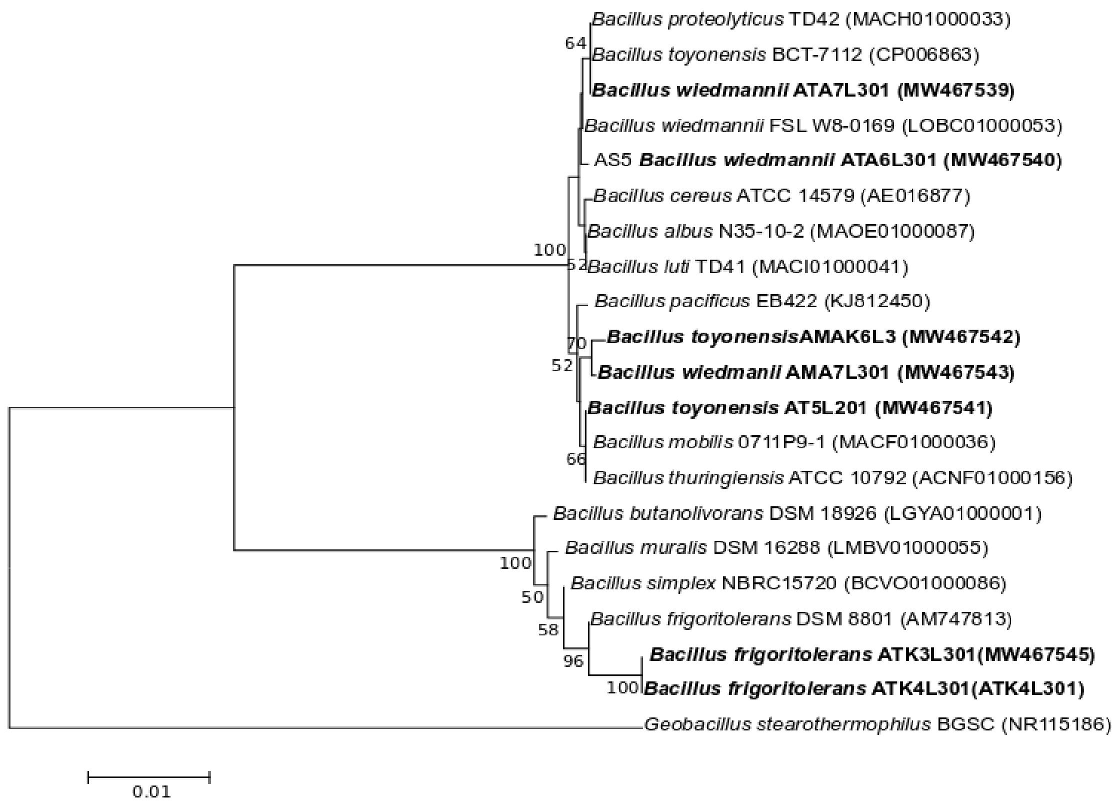
Bacillus proteolyticus TD42 (MACH01000033)
Bacillus toyonensis BCT-7112 (CP006863)
Bacillus wiedmannii ATA7L301 (MW467539)
Bacillus wiedmannii FSL W8-0169 (LOBC01000053)
AS5 Bacillus wiedmannii ATA6L301 (MW467540)
Bacillus cereus ATCC 14579 (AE016877)
Bacillus albus N35-10-2 (MAOE01000087)
Bacillus luti TD41 (MACI01000041)
Bacillus pacificus EB422 (KJ812450)
Bacillus toyonensisAMAK6L3 (MW467542)
Bacillus wiedmanii AMA7L301 (MW467543)
Bacillus toyonensis AT5L201 (MW467541)
Bacillus mobilis 0711P9-1 (MACF01000036)
Bacillus thuringiensis ATCC 10792 (ACNF01000156)
Bacillus butanolivorans DSM 18926 (LGYA01000001)
Bacillus muralis DSM 16288 (LMBV01000055)
Bacillus simplex NBRC15720 (BCVO01000086)
Bacillus frigoritolerans DSM 8801 (AM747813)
Bacillus frigoritolerans ATK3L301(MW467545)
Bacillus frigoritolerans ATK4L301(ATK4L301)
Geobacillus stearothermophilus BGSC (NR115186)
64
100
52
70
52
66
100
50
58
96
100
0.01

B)

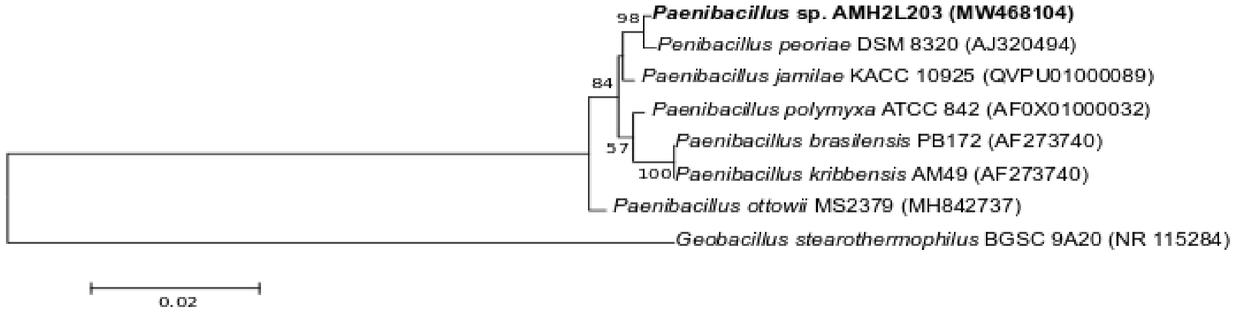
Paenibacillus sp. AMH2L203 (MW468104)
Penibacillus peoriae DSM 8320 (AJ320494)
Paenibacillus jamilae KACC 10925 (QVPU01000089)
Paenibacillus polymyxa ATCC 842 (AF0X01000032)
Paenibacillus brasilensis PB172 (AF273740)
Paenibacillus kribbensis AM49 (AF273740)
Paenibacillus ottowii MS2379 (MH842737)
Geobacillus stearothermophilus BGSC 9A20 (NR 115284)
98
84
57
100
0.02

C)

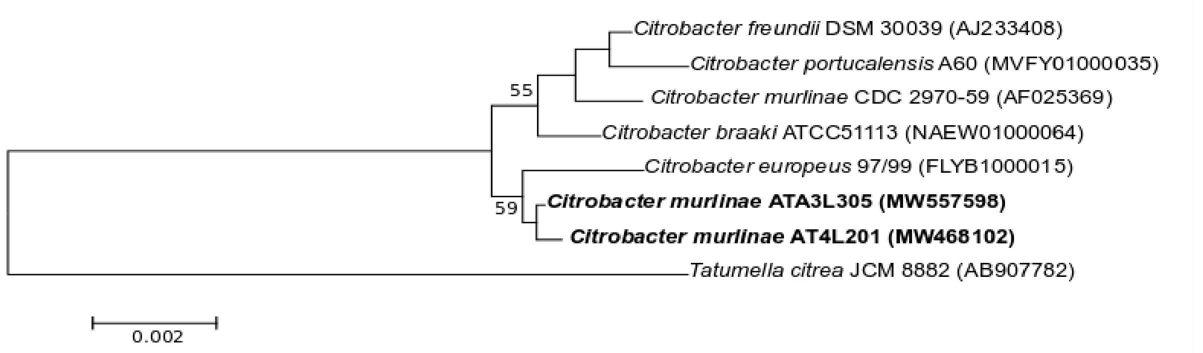
Citrobacter freundii DSM 30039 (AJ233408)
Citrobacter portucalensis A60 (MVFY01000035)
Citrobacter murlinae CDC 2970-59 (AF025369)
Citrobacter braaki ATCC51113 (NAEW01000064)
Citrobacter europeus 97/99 (FLYB1000015)
Citrobacter murlinae ATA3L305 (MW557598)
Citrobacter murlinae AT4L201 (MW468102)
Tatumella citrea JCM 8882 (AB907782)
55
59
0.002

D)

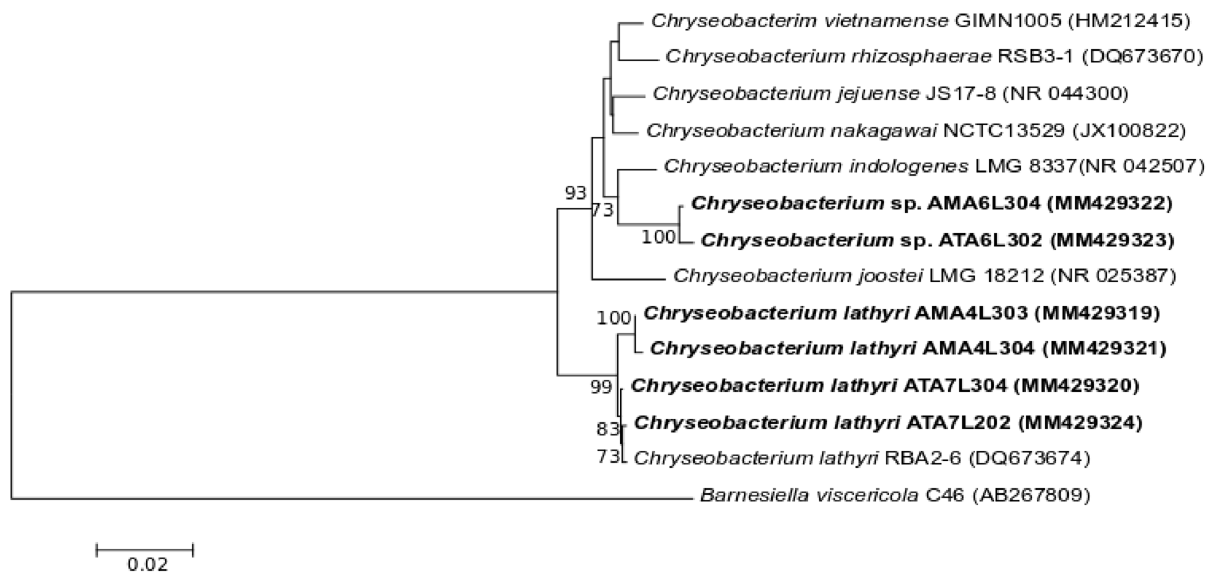
Chryseobacterim vietnamense GIMN1005 (HM212415)
Chryseobacterium rhizosphaerae RSB3-1 (DQ673670)
Chryseobacterium jejuense JS17-8 (NR 044300)
Chryseobacterium nakagawai NCTC13529 (JX100822)
Chryseobacterium indologenes LMG 8337(NR 042507)
Chryseobacterium sp. AMA6L304 (MM429322)
Chryseobacterium sp. ATA6L302 (MM429323)
Chryseobacterium joostei LMG 18212 (NR 025387)
Chryseobacterium lathyri AMA4L303 (MM429319)
Chryseobacterium lathyri AMA4L304 (MM429321)
Chryseobacterium lathyri ATA7L304 (MM429320)
Chryseobacterium lathyri ATA7L202 (MM429324)
Chryseobacterium lathyri RBA2-6 (DQ673674)
Barnesiella viscericola C46 (AB267809)
93
73
100
100
99
83
73
0.02

E)

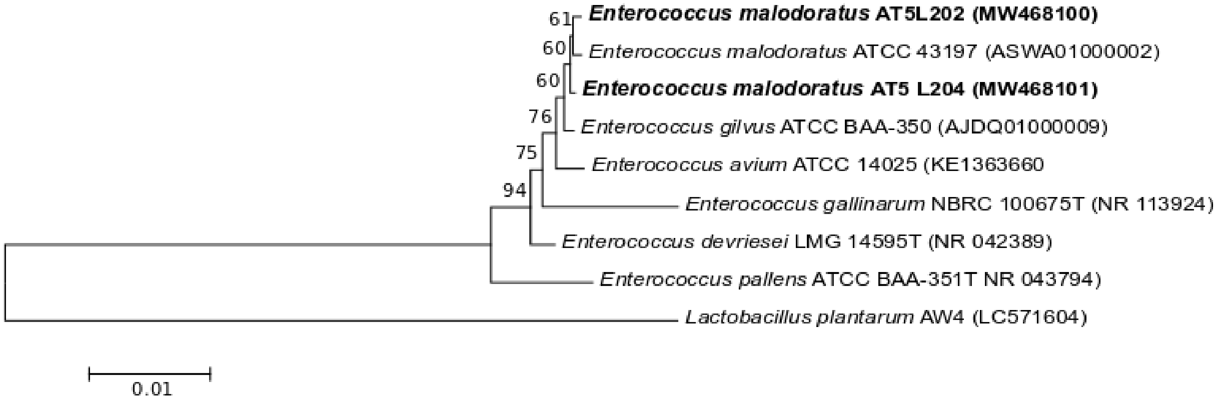

F)

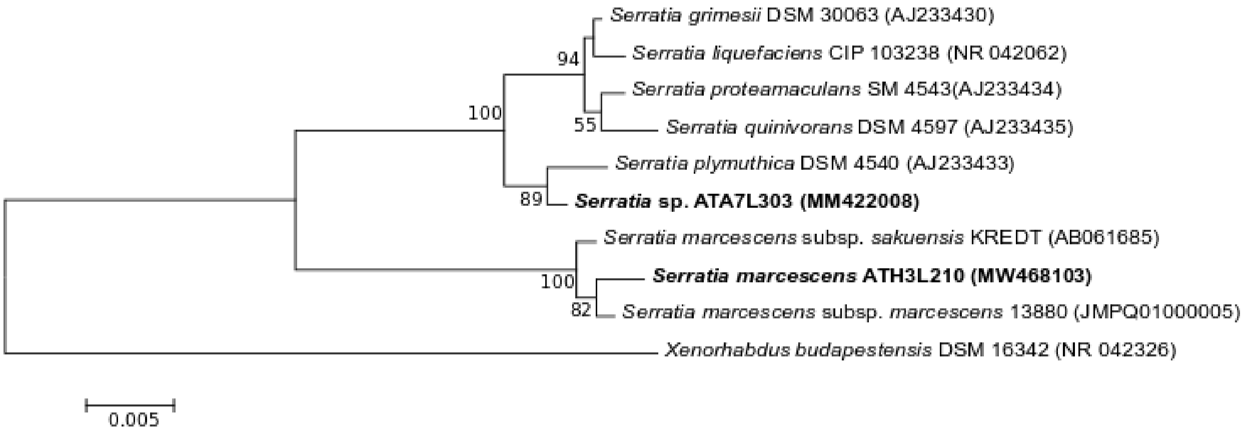

Figure S1. Phylogenetic relationships among representative *Amphimallon solstitile* midgut isolates and type strains of bacteria belonging to the genera: A) *Bacillus*; B) *Paenibacillus*; C) *Citrobacter*; D) *Chryseobacterium*; E) *Enterococcus*; F) *Serratia* based on 16S rRNA gene sequence analysis neighbor-joining method. The GenBank accession numbers are given in parenthesis. Bootstrap values ≥50% are displayed at branching points. The sequences generated from this study are listed in bold.

A)

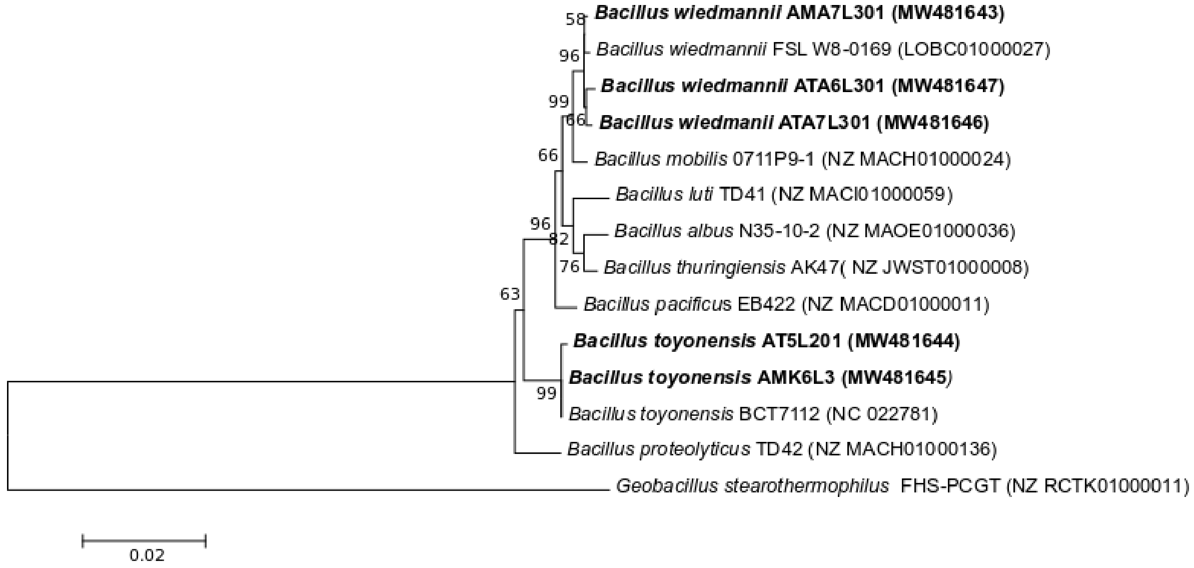

Bacillus wiedmannii AMA7L301 (MW481643)
Bacillus wiedmannii FSL W8-0169 (LOBC01000027)
Bacillus wiedmannii ATA6L301 (MW481647)
Bacillus wiedmanii ATA7L301 (MW481646)
Bacillus mobilis 0711P9-1 (NZ MACH01000024)
Bacillus luti TD41 (NZ MACI01000059)
Bacillus albus N35-10-2 (NZ MAOE01000036)
Bacillus thuringiensis AK47(NZ JWST01000008)
Bacillus pacificus EB422 (NZ MACD01000011)
Bacillus toyonensis AT5L201 (MW481644)
Bacillus toyonensis AMK6L3 (MW481645)
Bacillus toyonensis BCT7112 (NC 022781)
Bacillus proteolyticus TD42 (NZ MACH01000136)
Geobacillus stearothermophilus FHS-PCGT (NZ RCTK01000011)
58
96
99
66
66
96
82
76
63
99
0.02

B)

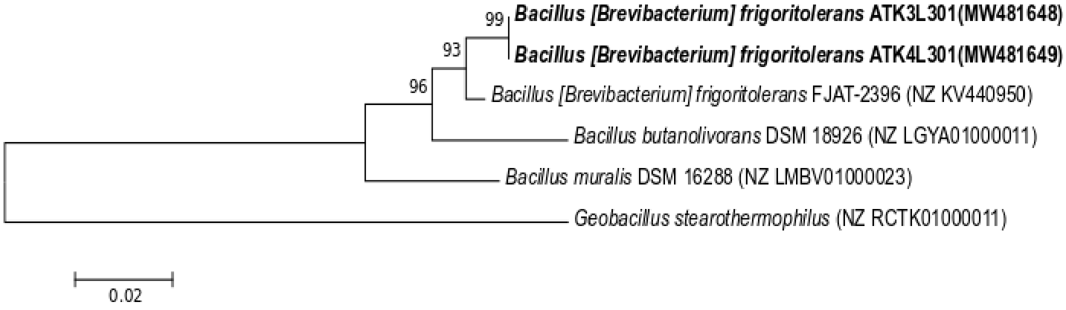

Bacillus [Brevibacterium] frigoritolerans ATK3L301(MW481648)
Bacillus [Brevibacterium] frigoritolerans ATK4L301(MW481649)
Bacillus [Brevibacterium] frigoritolerans FJAT-2396 (NZ KV440950)
Bacillus butanolivorans DSM 18926 (NZ LGYA01000011)
Bacillus muralis DSM 16288 (NZ LMBV01000023)
Geobacillus stearothermophilus (NZ RCTK01000011)
99
93
96
0.02

C)

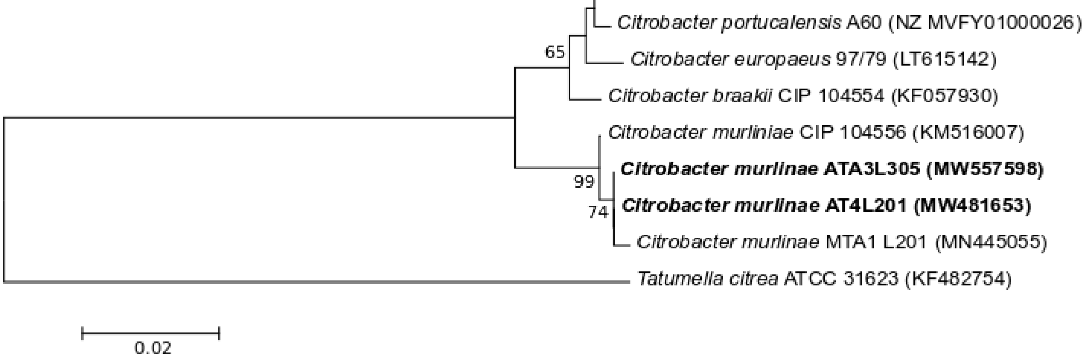

Citrobacter freundii ATCC 8090 (BBMV01000034)
Citrobacter portucalensis A60 (NZ MVFY01000026)
Citrobacter europaeus 97/79 (LT615142)
Citrobacter braakii CIP 104554 (KF057930)
Citrobacter murliniae CIP 104556 (KM516007)
Citrobacter murlinae ATA3L305 (MW557598)
Citrobacter murlinae AT4L201 (MW481653)
Citrobacter murlinae MTA1 L201 (MN445055)
Tatumella citrea ATCC 31623 (KF482754)
51
65
99
74
0.02

D)

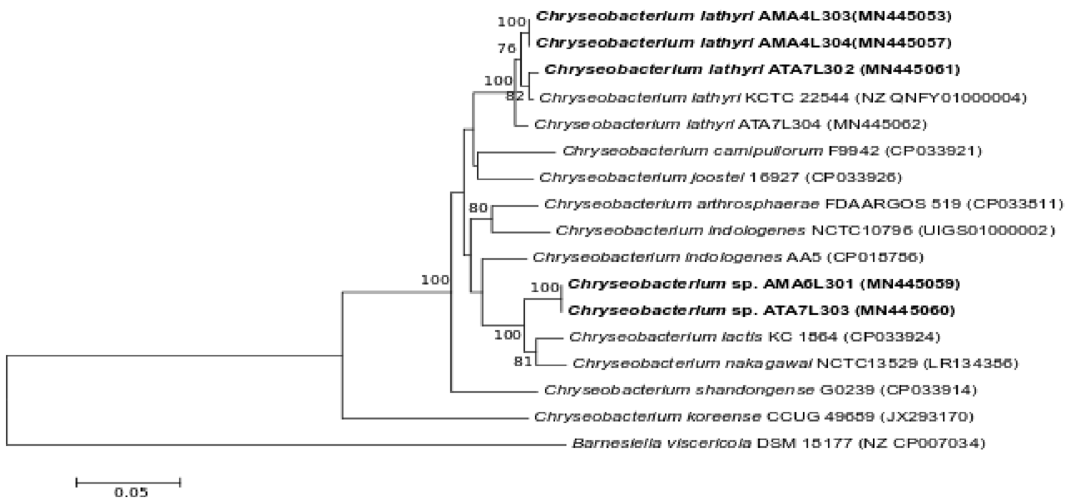
100
Chryseobacterium lathyri AMA4L303(MN445053)
76
Chryseobacterium lathyri AMA4L304(MN445057)
100
Chryseobacterium lathyri ATA7L302 (MN445061)
82
Chryseobacterium lathyri KCTC 22544 (NZ QNFY01000004)
Chryseobacterium lathyri ATA7L304 (MN445062)
Chryseobacterium camipullorum F9942 (CP033921)
Chryseobacterium joostei 16927 (CP033926)
80
Chryseobacterium arthrosphaerae FDAARGOS 519 (CP033811)
Chryseobacterium indologenes NCTC10796 (UIGS01000002)
Chryseobacterium indologenes AA5 (CP015756)
100
100
Chryseobacterium sp. AMA6L301 (MN445059)
Chryseobacterium sp. ATA7L303 (MN445060)
100
Chryseobacterium lactis KC 1864 (CP033924)
81
Chryseobacterium nakagawai NCTC13529 (LR134386)
Chryseobacterium shandongense G0239 (CP033914)
Chryseobacterium koreense CCUG 49689 (JX293170)
Barnesiella viscericola DSM 18177 (NZ CP007034)
0.05

E)

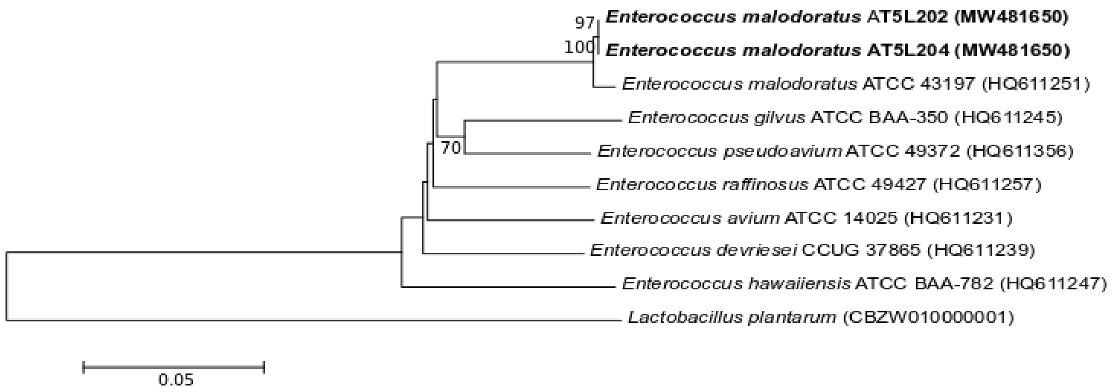
97
Enterococcus malodoratus AT5L202 (MW481650)
100
Enterococcus malodoratus AT5L204 (MW481650)
Enterococcus malodoratus ATCC 43197 (HQ611251)
Enterococcus gilvus ATCC BAA-350 (HQ611245)
70
Enterococcus pseudoavium ATCC 49372 (HQ611356)
Enterococcus raffinosus ATCC 49427 (HQ611257)
Enterococcus avium ATCC 14025 (HQ611231)
Enterococcus devriesei CCUG 37865 (HQ611239)
Enterococcus hawaiiensis ATCC BAA-782 (HQ611247)
Lactobacillus plantarum (CBZW010000001)
0.05

F)

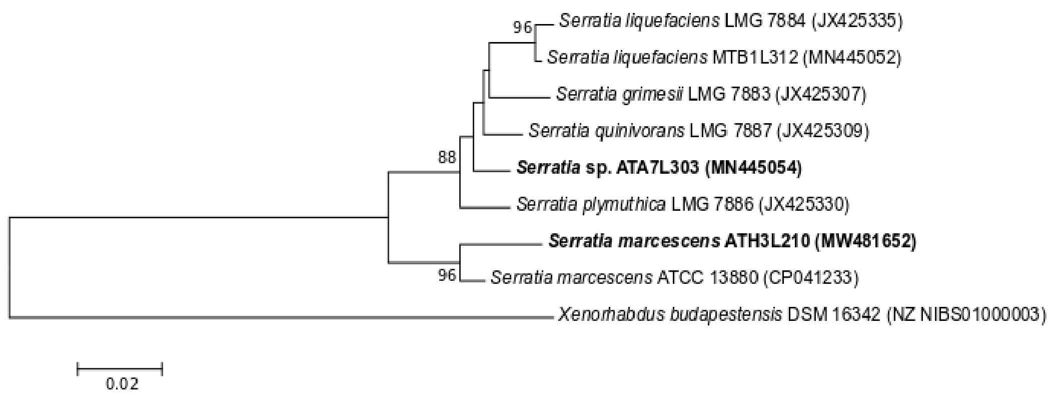

Figure S2. Phylogenetic relationships among representative *Amphimallon solstitile* midgut isolates and type strains of bacteria belonging to the genera: A, B) *Bacillus*; C) *Citrobacter*; D) *Chryseobacterium*; E) *Enterococcus*; F) *Serratia* based on *rpoB* gene sequence analysis with the neighbor-joining method. The GenBank accession numbers are given in parenthesis. Bootstrap values ≥50% are displayed at branching points. The sequences generated from this study are listed in bold.

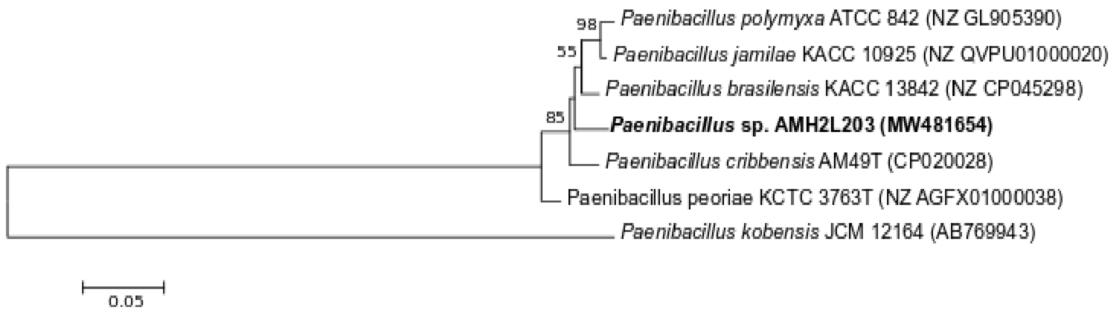

Figure S3. Phylogenetic relationships among representative *Amphimallon solstitile* midgut isolates and type strains of bacteria belonging to the genus *Paenibacillus* based on *gapA* gene sequence analysis with the neighbor-joining method. The GenBank accession numbers are given in parenthesis. Bootstrap values ≥50% are displayed at branching points. The sequences generated from this study are listed in bold.
